# Identification of exosomal ceRNA networks as prognostic markers in clear cell renal cell carcinoma

**DOI:** 10.1097/MD.0000000000040167

**Published:** 2024-10-25

**Authors:** Tao Zhu, Haizhu Fu, Zhiqiang Wang, Shanchun Guo, Shidong Zhang

**Affiliations:** a Department of Urology, Weifang People’s Hospital, Weifang, Shandong Province, China; b Department of Urology, Shouguang Hospital of Traditional Chinese Medicine, Shouguang, Shandong Province, China; c RCMI Cancer Research Center, Xavier University of Louisiana, New Orleans, LA.

**Keywords:** circRNA, lncRNA, miRNA, mRNA, prognostic marker, renal cell carcinoma

## Abstract

Aggressive clear cell renal cell carcinoma (ccRCC) has a bad prognosis. We seek new ccRCC biomarkers for diagnosis and treatment. We used exoRBase and The Cancer Genome Atlas Database to compare DEmRNAs, DEmiRNAs, DElncRNAs, and DEcircRNAs in ccRCC and normal renal tissues. CircRNAs and circRNAs targeting microRNAs (miRNAs) were anticipated and taken intersections, and several databases assessed the targeted link between common miRNAs and messenger RNAs (mRNAs). The Cancer Genome Atlas database was used to create a predictive mRNA signature that was validated in E-MTAB-1980. Finally, we examined competing endogenous RNA network miRNAs and long noncoding RNAs for ccRCC predictive biomarkers using overall survival analysis. We built the first competing endogenous RNA regulation network of circRNA–lncRNA–miRNA–mRNA and found that it substantially correlates with ccRCC prognosis. We unveiled ccRCC’s posttranscriptional regulation mechanism in greater detail. Our findings identified novel biomarkers for ccRCC diagnosis, therapy, and prognosis.

## 1. Introduction

Renal cell carcinoma (RCC) mainly originates from renal tubular epithelial cells and accounts for about 2% to 3% of all adult tumors.^[1]^ In developed countries, the incidence of RCC is increasing at a rate of 2% per year.^[2]^ Clear cell renal cell carcinoma (ccRCC) accounting for 80% to 90% of RCC is a common histopathologic subtype of kidney cancer.^[3,4]^ Both a higher body mass index and hypertension have been demonstrated to independently enhance the chance of developing RCC over the long run.^[5]^ Although surgical resection is the standard treatment for localized tumors, up to 40% of patients still experience disease recurrence, mainly in the first 5 years after surgery.^[6]^ ccRCC exhibits significant aggressiveness and is associated with a dismal prognosis; thus, there is an urgent want for precise biomarkers to facilitate clinical decision-making and prognostic evaluation, alongside the discovery of novel therapeutic agents.

In research involving 537 ccRCC patients, The Cancer Genome Atlas (TCGA) consortium identified notable abnormalities within the ccRCC group.^[7]^ The alterations encompass mutations in genes and unique configurations involving messenger RNA (mRNA) and microRNA (miRNA). These modifications indicate essential processes in ccRCC. Recent studies have identified significant functions for noncoding RNAs (ncRNAs), a category of RNAs that constitutes nearly 80% of the transcriptome.^[8,9]^ ncRNAs are a common set of RNAs that lack any protein-coding potentials.^[10]^ Circular RNA (circRNA) is a form of ncRNA characterized by a closed circular single-strand structure, consisting of hundreds to thousands of nucleotides, and lacking both a 5′ cap structure and a 3′ polyadenylated tail.^[11]^ Long noncoding RNA (lncRNA) is characterized as linear noncoding RNAs exceeding 200 nucleotides in length, capable of functioning as a sponge to sequester miRNA.^[12,13]^ CircRNAs and mRNAs possess miRNA-responsive element sequences that can bind miRNAs, leading to competition for scarce miRNAs and the establishment of a competitive endogenous RNA (ceRNA) regulation network. The ceRNA network is crucial in various physiological and pathological processes.^[14]^ The aberrant ceRNA network influences the initiation and progression of multiple tumor types.^[15,16]^

Exosomes are a subset of extracellular vesicles generated by all cells and are important for cell signal transduction.^[17,18]^ They transfer proteins, RNAs, and lipids to recipient cells, thus participating in various biological and pathological processes.^[19–22]^ Exosomes have been examined in all body fluids^[23]^ and exosomal RNA has been identified in various tumors,^[24]^ indicating that exosomal RNAs could be used as biomarkers for diagnosis and treatment.^[25–27]^ Exosomes can help tumor cell initiation, proliferation, metastasizing, and developing drug resistance.^[28–30]^ Several studies recently reported that exosomes have significant differences between ccRCC patients and healthy controls.^[31,32]^ It plays a critical role in the metastasis of ccRCC,^[33]^ and participates in the process of tumor drug resistance and immune escape.^[34,35]^ A few studies reported the prognostic value of lncRNA–miRNA–mRNA^[36]^ and circRNA–miRNA–mRNA^[37]^ ceRNA network in ccRCC. Nevertheless, little is known about the functions of exosomes or exosomal circRNA–lncRNA–miRNA–mRNA regulatory networks in ccRCC.

This study utilized exoRBase and the TCGA Database to evaluate and identify differentially expressed mRNAs, miRNAs, long noncoding RNAs (lncRNAs), and circRNAs between ccRCC and normal tissues, thereby ensuring the correctness and reproducibility of the analytical data. miRNAs targeted by lncRNAs and circRNAs were independently predicted utilizing several databases. Then, we established and validated a prognostic mRNA signature. Finally, we developed a new strategy for the prognosis and treatment of ccRCC after processing an overall survival (OS) analysis on miRNAs and lncRNAs in the ceRNA network.

## 2. Materials and methods

### 2.1. Data collection and preprocessing

For identifying some potential functional ceRNAs in the initiation and progression of kidney renal clear cell carcinoma (KIRC), we used the exoRBase database (http://www.exorbase.org/). This database contains circRNAs, lncRNAs, and mRNAs derived from RNA-seq data analyses in different human body fluids. In this study, the mRNA expression profiles, which included 15 KIRC and 118 healthy blood samples, were obtained from the exoRBase database up to May 1, 2022.

From the TCGA database (https://portal.gdc.cancer.gov/), RNA profiles including HTseq-counts of 541 KIRC and 72 normal kidney tissue samples were downloaded. Clinical data for 537 KIRC samples, encompassing gender, age, pathological stage, AJCC TNM stage, and prognostic information, was also acquired. Then, the somatic mutation data was downloaded and the tumor mutation burden (TMB) per megabase of each sample was also determined. We downloaded the E-MTAB-1980 cohort (https://www.ebi.ac.uk/arrayexpress/) as an external validation cohort. A flowchart (Fig. S1, Supplemental Digital Content, http://links.lww.com/MD/N810) was incorporated to encapsulate the procedures.

### 2.2. Differential expression analysis in exoRBase and TCGA KIRC

We used the Limma and edgeR packages to analyze differentially expressed circRNAs (DEcircRNAs), lncRNAs (DElncRNAs), and mRNAs (DEmRNAs) between healthy and KIRC blood samples. mRNAs and lncRNAs were also analyzed by using the Ensembl database. Abnormally expressed miRNAs (DEmiRNAs), DElncRNAs, and DEmRNAs between KIRC and adjacent normal samples were identified by using the edgeR package in R. | logFC| > 1 and adj. *P* value < .05 was considered as significance.^[38,39]^ We used the ggplot2 package in R to obtain volcano plots and heatmaps.

### 2.3. The construction of the ceRNA regulatory network

Increasing data reveals that some circRNAs and lncRNAs act as “sponges” to trap miRNAs in malignancies. The concept that circRNAs and lncRNAs operate as miRNA sponges to directly interact with and regulate mRNA activity led to the creation of the circRNA–lncRNA–miRNA–mRNA ceRNA network. circBase (http://www.circbase.org/) has circRNA information.^[40]^ The cancer-specific circRNA database (http://gb.whu.edu.cn/CircView/) predicted target miRNAs.^[41]^ The starBase database (https://starbase.sysu.edu.cn/) analyzes lncRNA target miRNAs.^[42]^ TCGA further screened these target miRNAs. In the next step, miRTarBase, TargetScan, and miRDB predicted specific miRNAs. Finally, screening the circRNA–miRNA, lncRNA–miRNA, and miRNA–mRNA groups created the circRNA–lncRNA–miRNA–mRNA regulatory network. The data were visualized with Cytoscape 3.8.0.

### 2.4. ceRNA network: construction and validation of Cox proportional hazards regression model

The TCGA cohort samples were designated as the training set, whilst the E-MTAB-1980 samples were designated as the test set. First, we identified candidate target mRNAs that were differentially expressed in TCGA and normalized the expression of DEmRNA according to [log2(data + 1)] before further analysis. Prognostic mRNAs were identified from DEmRNAs using univariate Cox regression analysis in the training set. The genes with a *P*-value <.05 were selected. “glmnet” R package was used to further process the prognosis-related mRNAs using LASSO Cox regression analysis to generate a prognostic risk score model for predicting OS of KIRC samples. The 10-fold cross-validation was conducted to determine the penalty parameter (λ) of the model. The formula below was used to calculate each sample risk score.


$$\begin{array}{l} The\;risk\;score=\underset{i=1}{\overset{n}{\sum }}\;Coef_{i}\times Expr_{i} \end{array}$$

Expr_*i*_ denotes the expression values of gene *i* from the prognostic risk score model, while coef_*i*_ signifies the regression coefficient of gene *i* within the signature. Based on the median value of risk scores, all samples were divided into low- and high-risk score groups. Kaplan–Meier analysis was used to compare the OS difference between the above 2 groups. The time-dependent receiver operating characteristic (ROC) curves were plotted using the “timeROC” package in R to determine the predictive accuracy of the model. Univariate and multivariate Cox regression analyses were used to analyze the independence and prognostic value of the model. Last step, the prognostic risk score model was further validated in the test set for its reliability and applicability.

### 2.5. Tumor-infiltrating immune cells: CIBERSORT estimation and survival analysis

For the purpose of analyzing the level of immune cells in the 2 groups and the connection between immune cells and key biomarkers in the ceRNA network, we estimated the proportion of 22 kinds of immune cell types in 541 tumor tissues and 72 normal adjacent tissues by CIBERSORT, an algorithm for characterizing cell composition of complex tissues from their gene expression profiles. We only included the samples with CIBERSORT output of *P* < .05 for further analysis. After screening samples, we searched for immune cells with significant differences between cancer tissues and normal adjacent tissues, as indicated by a Wilcoxon rank-sum test. The correlation between the composition of various immune cells and the overall survival of KIRC patients was assessed by Kaplan–Meier survival curve analysis.

### 2.6. Tumor-infiltrating immune cells: Cox proportional hazards regression model

Immune cells with significant effects were first identified using univariate Cox analysis, then results were further tested using the initial Cox model and the Lasso regression. Based on the multivariate model of immune cells, using ROC and calibration curves, we constructed and confirmed the discrimination and precision of the nomogram. We divided the patients into high and low-risk groups according to the median risk score using K-M survival curve analysis. Finally, the relationship between immune cells and genes using Pearson correlation analysis.

### 2.7. Protein levels of prognostic model genes in the Human Protein Atlas database

For the purpose of mapping all the human proteins in cells, tissues, and organs, the Human Protein Atlas database (https://www.proteinatlas.org/) was used. The Human Protein Atlas database consists of 6 separate parts. Among that, the Tissue and the Pathology Atlas provide information regarding the expression profiles of specified genes in normal and tumor tissues on protein levels.

### 2.8. Statistical analysis

Statistical analyses were conducted utilizing GraphPad Prism 8 (GraphPad Software, Inc., San Diego, CA). A *P*-value <.05 was deemed statistically significant. The differential expression of mRNAs and miRNAs was assessed utilizing the limma R packages (version 4.1.3) from the University of Melbourne, Parkville, Victoria, Australia. A *P*-value <.05 and an FDR <.05 were deemed statistically significant. Differential studies of lncRNA and circRNA expression were conducted utilizing the limma R package (4.1.3) from the University of Melbourne. A *P*-value <.05 was deemed statistically significant.

## 3. Results

### 3.1. Identification of differentially expressed circRNAs, lncRNAs, mRNAs, and miRNAs

**Figure 1.**
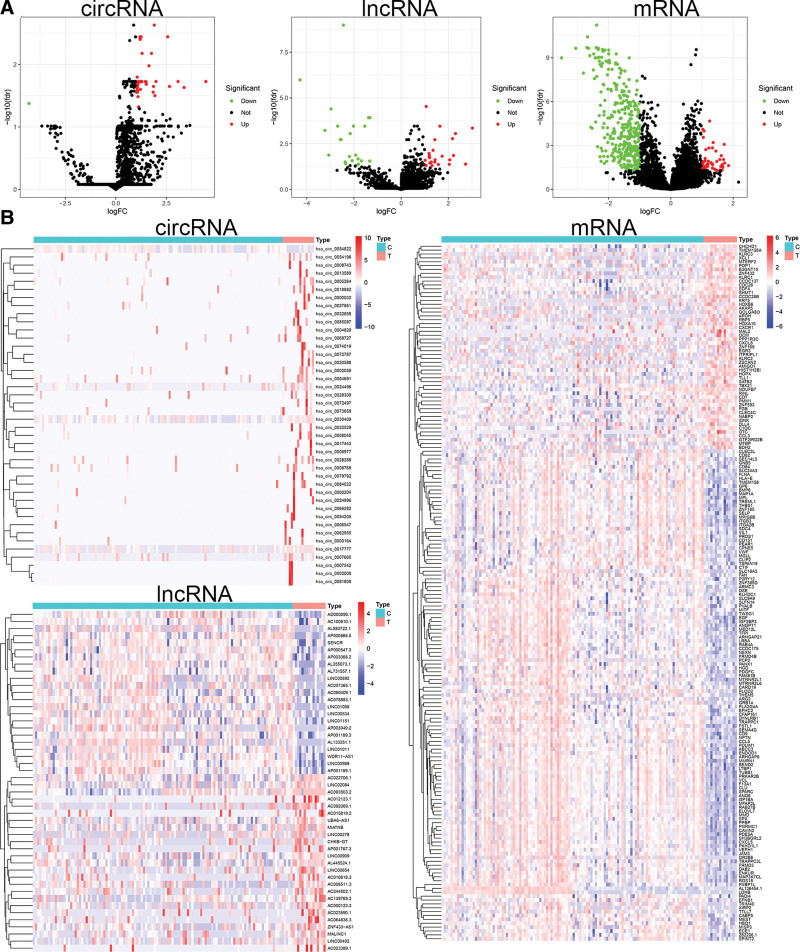
Identification of differentially expressed RNAs (circRNAs, lncRNAs, and mRNAs) in healthy and KIRC blood samples. (A) Volcano plots from healthy and KIRC blood samples demonstrating circRNA, lncRNA, and mRNA expression differences. Green and red denote downregulated and upregulated. (B) Heatmaps show circRNA, lncRNA, and mRNA expression differences between healthy and KIRC blood samples. Normal samples are blue, and KIRC samples red. KIRC = kidney renal clear cell carcinoma, lncRNAs = long noncoding RNAs, mRNA = messenger RNA.

**Figure 2.**
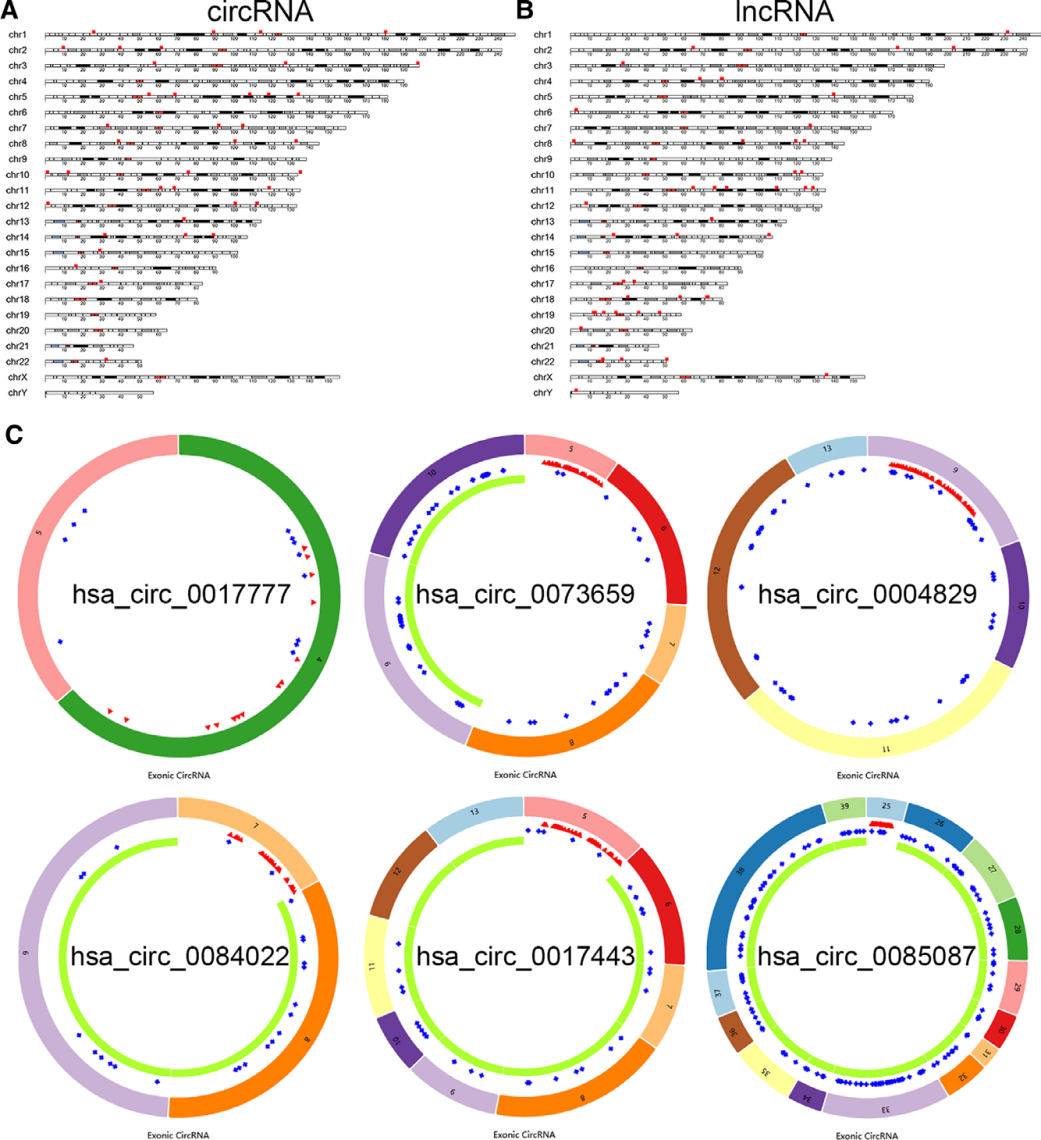
The chromosome map of DEcircRNAs and DElncRNAs and structural patterns of circRNAs. (A) DEcircRNA chromosomal map in healthy and KIRC blood samples. (B) DElncRNA chromosomal map in healthy and KIRC blood samples. The width of the bar denotes RNA length. (C) The 6 circRNAs’ basic structure. Red is MRE, blue is RBP, and green is ORF. KIRC = kidney renal clear cell carcinoma, lncRNAs = long noncoding RNAs, MRE = miRNA-responsive element, ORF = open reading frame, RBP = RNA binding protein.

To find some potential ceRNA associated with carcinogenesis and progression of KIRC, 15 KIRC and 118 healthy blood samples from the exoRBase database were finally used, and differential expression analysis was performed on healthy and KIRC blood samples to identify DEcircRNA, DElncRNA, and DEmiRNA. Meanwhile, we analyzed the DEmiRNAs, DElncRNAs, and DEmRNAs in KIRC and normal kidney tissues with adj. *P* value < .05 and |logFC| > 1 as the thresholds using the TCGA database. After the analysis, a total of 42 DEcircRNAs (41 up- and 1 down-regulated), 48 DElncRNAs (25 up-, and 23 down-regulated), and 405 DEmRNAs (53 up- and 352 down-regulated) were found between healthy and KIRC blood samples. DEcircRNAs, DElncRNAs, and DEmRNAs were distributed in volcanic plots in Figure 1A. Figure 1B showed the heat map showing KIRC and normal sample expression profiles separated and consistent. We also compared DEmiRNAs, DElncRNAs, and DEmRNAs in 541 TCGA-KIRC and 72 adjacent-normal samples. Differentially expressed RNAs in KIRC included 48 lncRNAs (34 up- and 14 down-regulated), 211 miRNAs (115 up- and 96 down-regulated), and 1324 mRNAs (889 up- and 435 down-regulated) (Fig. S2, Supplemental Digital Content, http://links.lww.com/MD/N810). The chromosome map showed the source gene positions and lengths of DEcircRNAs (Fig. 2A) and DElncRNAs (Fig. 2B). Figure 2C revealed the 6 circRNAs’ basic structure. Six circRNA-miRNA interactions were found, including hsa_circ_0004829-miR-142-5p, 0085087-miR-142-5p, 0017777-miR-149-5p, 0073659-miR-149-5p, 0084022-miR-149-5p, and 0017443-miR-342-5p.

### 3.2. The development of the ceRNA regulatory network

**Figure 3.**
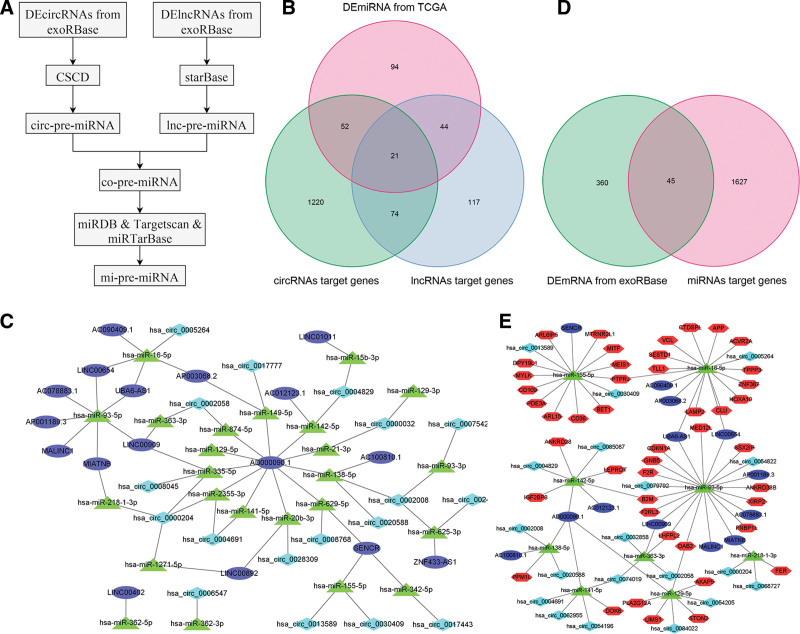
Illustrating the prediction of frequently targeted miRNAs, their corresponding differentially expressed mRNAs (DEmRNAs), and the competing endogenous RNA (ceRNA) network. (A) Flowchart illustrating the prediction of common pre-miRNAs. (B) Venn diagram illustrating the intersection of circ-pre-miRNAs, lnc-pre-miRNAs, and DEmiRNAs. (C) The correlation among differentially expressed circRNAs, differentially expressed lncRNAs, and their corresponding targeted miRNAs. (D) Venn diagram illustrating the intersection of mi-pre-mRNAs and DEmRNA. (E) CeRNA network comprising interactions among circRNA, lncRNA, miRNA, and mRNA. Cyan denotes circRNAs, blue signifies lncRNAs, red represents mRNAs, and green indicates miRNAs. lncRNAs = long noncoding RNAs, miRNA = microRNA, mRNA = messenger RNA.

Initially, we identified the miRNAs targeted by DEcircRNAs and DElncRNAs. Figure 3A illustrates a flowchart for the generation of often expected miRNAs. The target miRNAs of 48 differentially expressed lncRNAs were predicted utilizing the starBase database. The relationship between the 48 DElncRNAs and 256 miRNAs was discovered. A total of 42 DEcircRNAs and 1367 miRNAs with mutual interaction capabilities were found utilizing the cancer-specific circRNA database database. Figure 3B showed 21 common miRNAs targeted by DElncRNAs and DEcircRNAs, while Figure 3C revealed the connection between circRNAs, lncRNAs, and miRNAs. Then, 1952 potential target genes of 21 miRNAs were identified using the miRTarBase, TargetScan, and miRDB databases. Figure 3D shows the number of DEmRNA in the ceRNA network. According to the “ceRNA hypothesis,” we constructed a ceRNA regulatory network, by integrating the expression profiles and regulatory relationships of the lncRNAs, circRNAs, miRNAs, and mRNAs. A total of 19 DEcircRNAs and 9 DEmiRNAs were paired into 20 DEcircRNA–DEmiRNA interactions, 13 DElncRNAs, and 10 DEmiRNAs were paired into 20 DElncRNA–DEmiRNA interactions, whereas 10 DEmiRNAs and 45 DEmRNAs were matched to form 49 pairs of DEmiRNA–DEmRNA interactions. Finally, the KIRC-specific circRNA–lncRNA–miRNA–mRNA ceRNA regulatory network, which contained 86 nodes and 89 edges (Fig. 3E), was established. This study showed a thorough analysis of transcriptome data from the TCGA and exoRBase databases, leading to the development of a predictive circRNA–lncRNA–miRNA–mRNA ceRNA network for ccRCC patients.

### 3.3. Construction and validation of Cox proportional hazards regression model

**Figure 4.**
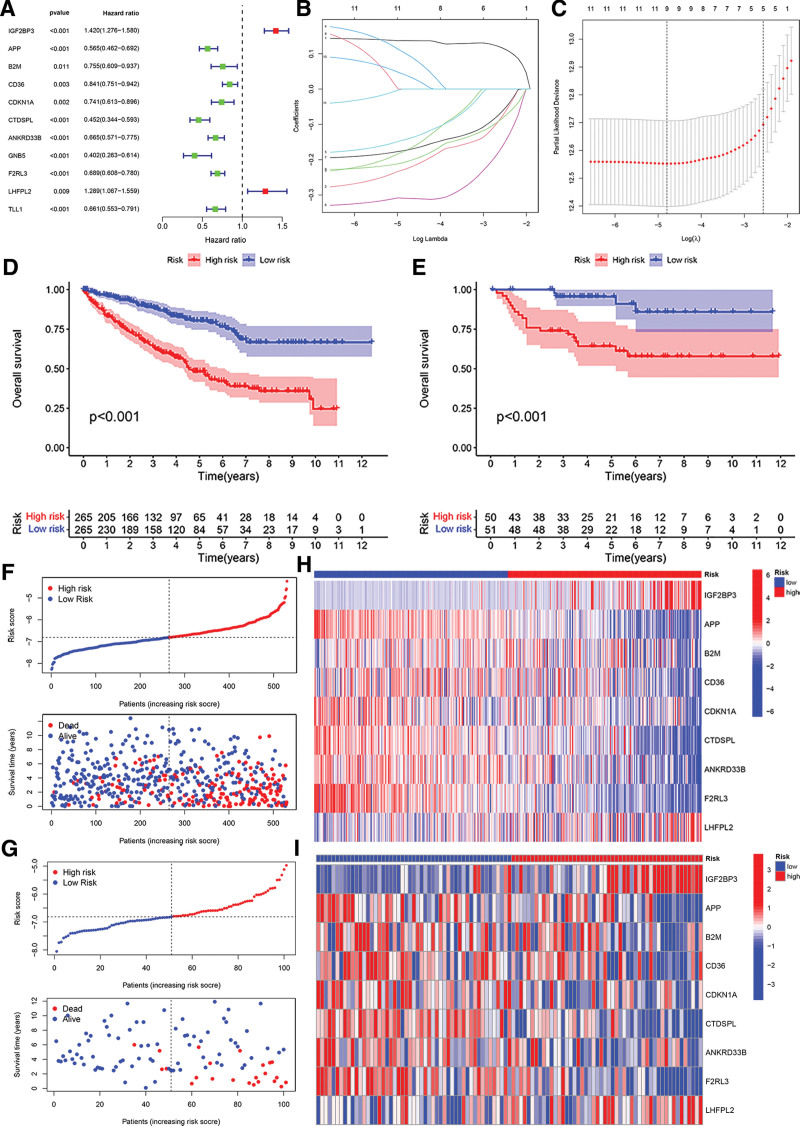
Prognostic analysis of the 9-gene signature model in the TCGA and E-MTAB-1980 cohort. (A) Forest plot of 11 prognostic potential target mRNAs who differ in expression. (B) The LASSO coefficient profile of 9 OS-related potential target mRNAs and perpendicular imaginary lines were drawn at the 10-fold cross-validation value. (C) The error curve was cross-verified by selecting tuning parameters (log λ) for OS-related putative target mRNAs. Perpendicular imaginary lines were drawn at the ideal value using the minimal and 1-se criteria. (D) TCGA cohort low- and high-risk score OS comparison. (E) E-MTAB-1980 low- and high-risk score OS comparison. (F) TCGA cohort 9-gene signature model risk score, survival status, and survival time. (G) E-MTAB-1980 cohort 9-gene signature model risk score, survival status, and survival period. (H) 9-gene signature model heatmap distribution for each TCGA cohort patient. (I) Heatmap of 9-gene signature model for each E-MTAB-1980 patient. TCGA = The Cancer Genome Atlas.

**Figure 5.**
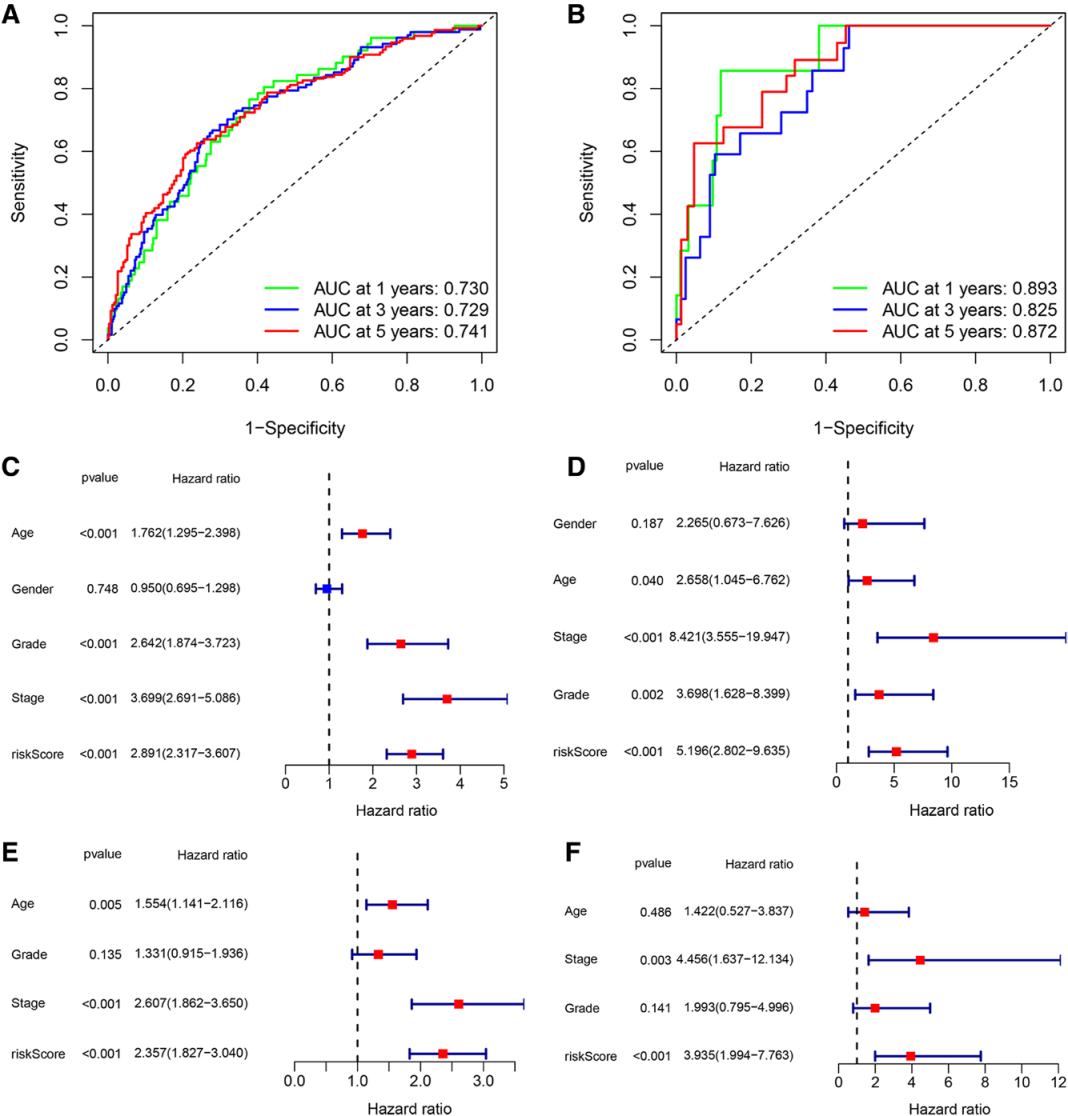
The association between clinicopathological factors and OS in the TCGA and E-MTAB-1980 cohort. (A) Time-dependent ROC curve AUC confirmed the risk score’s predictive efficacy in the TCGA cohort. (B) Time-dependent ROC curve AUC confirmed the risk score’s predictive efficacy in E-MTAB-1980. (C) Univariate Cox regression analyses of clinicopathological variables and OS in the TCGA cohort. (D) Univariate Cox regression analyses of clinicopathological variables and OS in E-MTAB-1980. E. TCGA cohort multivariate Cox regression analyses of clinicopathological variables and OS. (F) Multivariate Cox regression analyses of clinicopathological variables and OS in the E-MTAB-1980 cohort. OS = overall survival, ROC = receiver operating characteristic, TCGA = The Cancer Genome Atlas.

Some candidate target mRNAs (21/45) were aberrantly expressed between tumor tissues and normal tissues, and 11 of them were determined to correlate with OS in the univariate Cox regression analysis (Fig. 4A). Nine genes were identified as significantly correlated to survival based on the optimal value of λ (Fig. 4B and C). Subsequently, the TCGA–KIRC patients were divided into a high-risk or a low-risk group according to the median cutoff value. To test the prognostic capability, we evaluated the risk scores for every patient in the E-MTAB-1980 set and assigned them to high- and low-risk groups using the uniform formula. The test results were quite similar in the TCGA and E-MTAB-1980 cohort: KIRC patients with higher risk scores had a shorter OS time and lower OS rates (Fig. 4D and E). Figure 4F and G showed the risk score and survival status distributions which demonstrated the patients with higher risk scores had shorter OS time. The heatmap indicates that IGF2BP3 and LHFPL2 had elevated expression levels in the high-risk score group, while the low-risk score group demonstrated increased levels of APP, beta-2-microglobulin (B2M), CD36, CDKN1A, CTDSPL, ANKRD33B, and F2RL3 (Fig. 4H and I). The ROC analysis showed that the model possesses considerable prognostic value for KIRC patients in the TCGA cohort (1-year AUC = 0.730, 3-year AUC = 0.729, 5-year AUC = 0.741; Fig. 5A) and the E-MTAB-1980 cohort (1-year AUC = 0.893, 3-year AUC = 0.825, 5-year AUC = 0.872; Fig. 5B). The aforementioned results indicated that the model had a consistent and resilient capacity to predict overall survival. In the analysis using univariate Cox regression, the risk score was found to be significantly correlated with OS in both the TCGA and the E-MTAB-1980 cohort (HR = 2.891, 95% CI = 2.317–3.607, *P* < .001; HR = 5.196, 95% CI = 2.802–9.635, *P* < .001, respectively) (Fig. 5C and D). Upon the analysis using multivariate Cox regression, the risk score still proved to be an independent predictor for OS in the analysis (TCGA cohort: HR = 2.357, 95% CI = 1.827–3.040, *P* < .001; E-MTAB-1980 cohort: HR = 3.935, 95% CI = 1.994–7.763, *P* < .001; Fig. 5E and F), after correction for other confounding factors.

### 3.4. Tumor-infiltrating immune cells: CIBERSORT estimation and survival analysis

**Figure 6.**
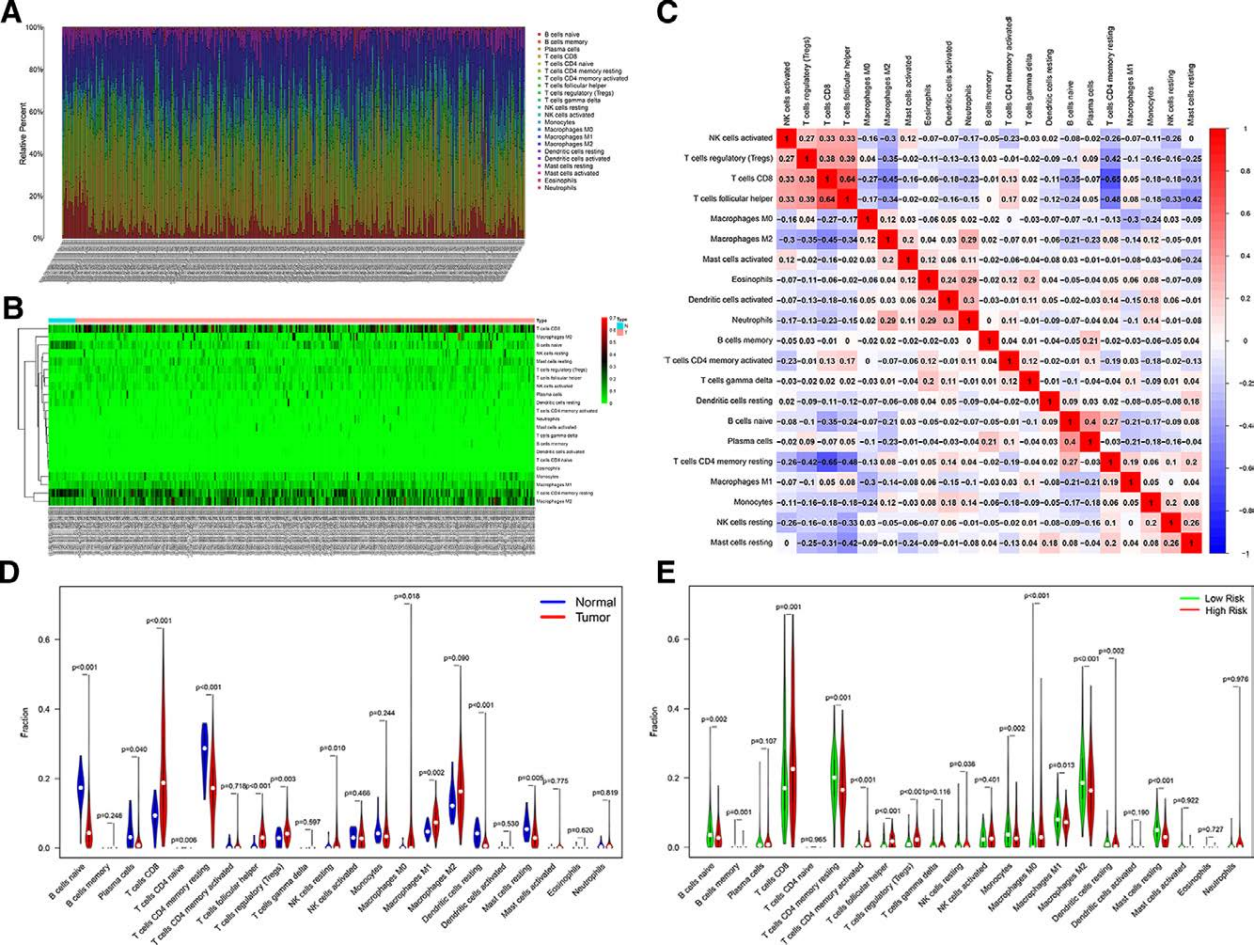
Tumor-infiltrating immune cells: CIBERSORT estimation and survival analysis. (A) Histogram depicting the infiltration ratios of 22 immune cell types within tumor tissue. (B) Heatmap illustrating the disparity in immune cell infiltration between cancerous tumors and neighboring normal tissue. (C) Correlation study of the expression levels of the 22 immune cell types in the tumor sample. (D) Violin plot illustrating the disparity in immune cell infiltration between cancerous tissue and neighboring normal tissue. (E) Violin plot illustrating the disparity in immune cell infiltration between low-risk and high-risk groups.

To investigate the disparity in tumor microenvironment between KIRC samples and normal samples, the prevalence of 22 types of immune cells was assessed. Figure 6A–C illustrated the proportion of 22 immune cell types utilizing the CIBERSORT method. The violin plot was generated using the Wilcoxon test. The findings indicated that the infiltration levels of CD8 T cells (*P* < .001), follicular helper T cells (*P* < .001), regulatory (Tregs) T cells (*P* = .003), resting NK cells (*P* = .010), M0 macrophages (*P* = .018), and M1 macrophages (*P* = .002) were higher in KIRC samples, while naive B cells (*P* < .001), plasma cells (*P* = .040), memory resting CD4 T cells (*P* < .001), resting dendritic cells (*P* < .001), and resting mast cells (*P* = .005) were significantly lower (Fig. 6D). To answer whether risk scores can act as immune indicators, we examined the correlation of prognostic risk scores with tumor-infiltrating immune cells. The findings indicated that the group exhibited a substantial presence of immune-suppressive cell infiltration, including Tregs and follicular helper T cells, aligning with the survival disadvantage observed in the high-risk cohort. Natural killer cells, including memory-activated CD4 T cells and CD8 T cells, were also noted to proliferate in the high-risk group (Fig. 6E).

### 3.5. Tumor-infiltrating immune cells: Cox proportional hazards regression model

**Figure 7.**
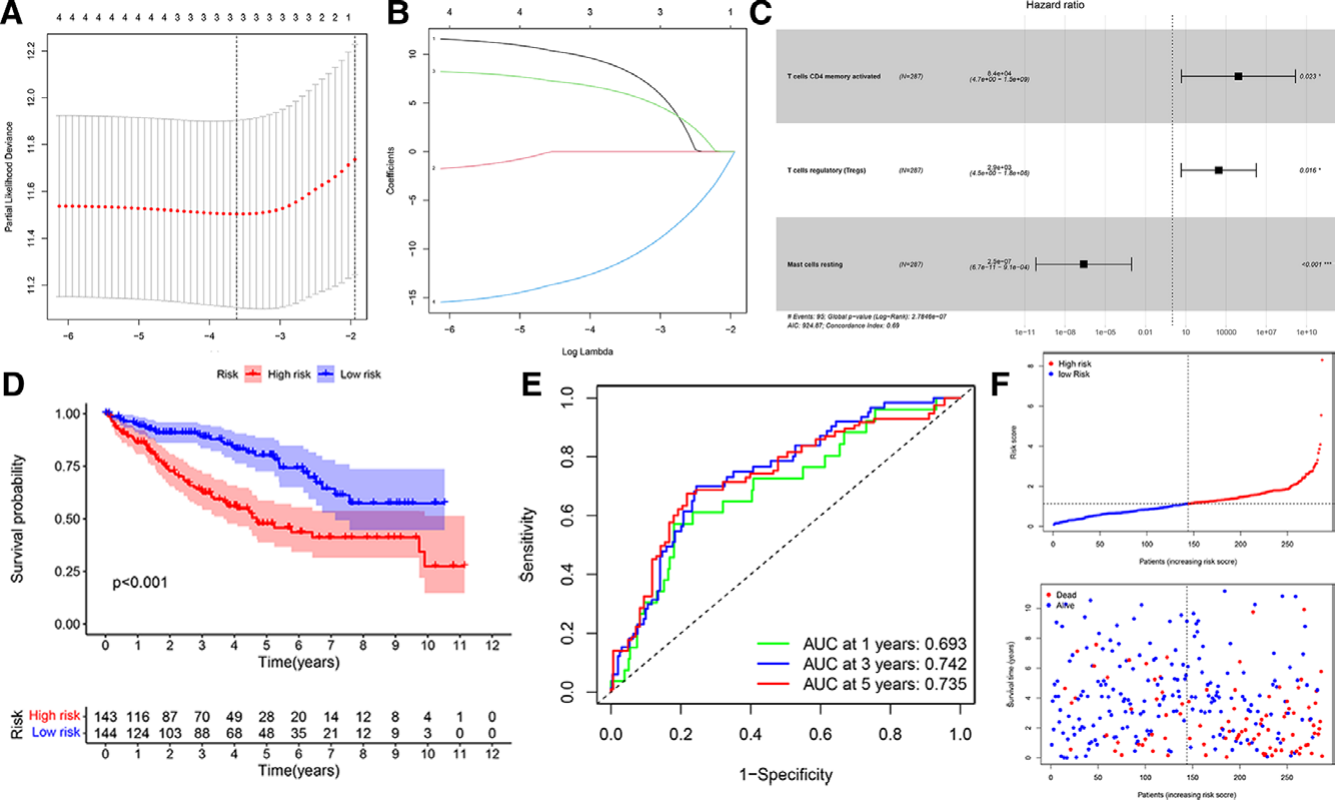
Tumor-infiltrating immune cells: Cox proportional hazards regression model. (A) Lasso regression analysis to screen immune cells related to prognosis. (B) Calculating the risk score of each patient and establishing the risk model. (C) Cox regression analysis of 3 prognostic-related immune cells. (D) High and low risk of immune cell model comparison of the prognosis of patients in the group. (E) The evaluation efficiency of the risk model at 1, 3, and 5 years. (F) Distribution of risk score, survival status, and survival time of 3 immune cells signature model.

**Figure 8.**
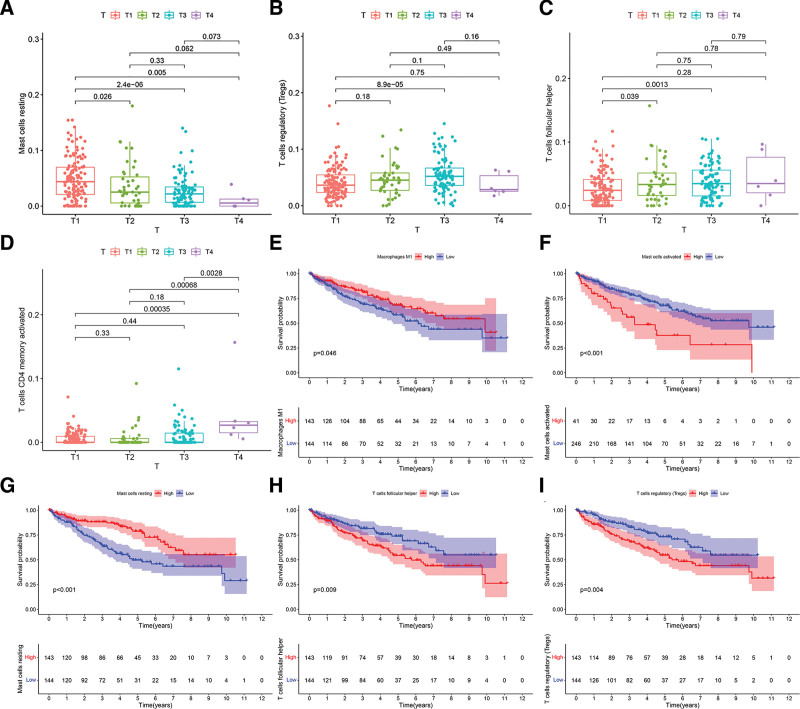
Correlation analysis between immune cells and clinical features. (A) Patient T1 to T4 resting mast cell expression. (B) Patient T1 to T4 regulatory (Tregs) T cell expression relations. (C) Patient T1 to T4 follicular helper T cell expression. (D) Memory-activated CD4 T cell expression in T1 to T4 patients. (E) Prognostic difference analysis of high- and low-M1 macrophage expression patients. (F) Prognostic difference study of highest and lowest resting mast cell expression patients. (G) Prognostic difference analysis of activated mast cell high and low expression patients. (H) Prognostic difference analysis of higher and lower follicular helper T cell expression. (I) Results of prognostic difference analysis of patients with high and low Treg T cell expression.

The immune cells with prognostic value were also examined using univariate Cox regression analysis. Three immune cells including follicular helper T cells, memory-activated CD4 T cells, and resting mast cells were detected using Lasso and multivariate Cox analyses (Fig. 7A–C). Based on the 3 immune cells, a new prognostic signature was established. The relative coefficients were derived by multivariate Cox regression analysis. The survival study indicated a poorer prognosis for patients in the high-risk group (Fig. 7D). ROC (AUCs of 1, 3, and 5-year survivals: 0.693, 0.742, and 0.735) curve indicated good prediction accuracy (Fig. 7E). The survival status distribution graph demonstrated that the number of deaths escalated with the rise in risk score (Fig. 7F). Additionally, we evaluated correlations between clinical and pathological factors and immune cells. The relative expression of the 22 kinds of immune cells in the T1-T4 stages of patients is shown in Figure 8A–D. We noted that patients exhibiting elevated levels of M1 macrophages and resting mast cells had a more favorable prognosis compared to those with reduced levels of M1 macrophages and resting mast cells. Patients exhibiting modest levels of activated mast cells and follicular helper T cells, as well as Tregs, demonstrated more favorable prognoses compared to those with high expression levels (Fig. 8E–I).

### 3.6. Co-expression analysis of immune cells and core mRNAs

**Figure 9.**
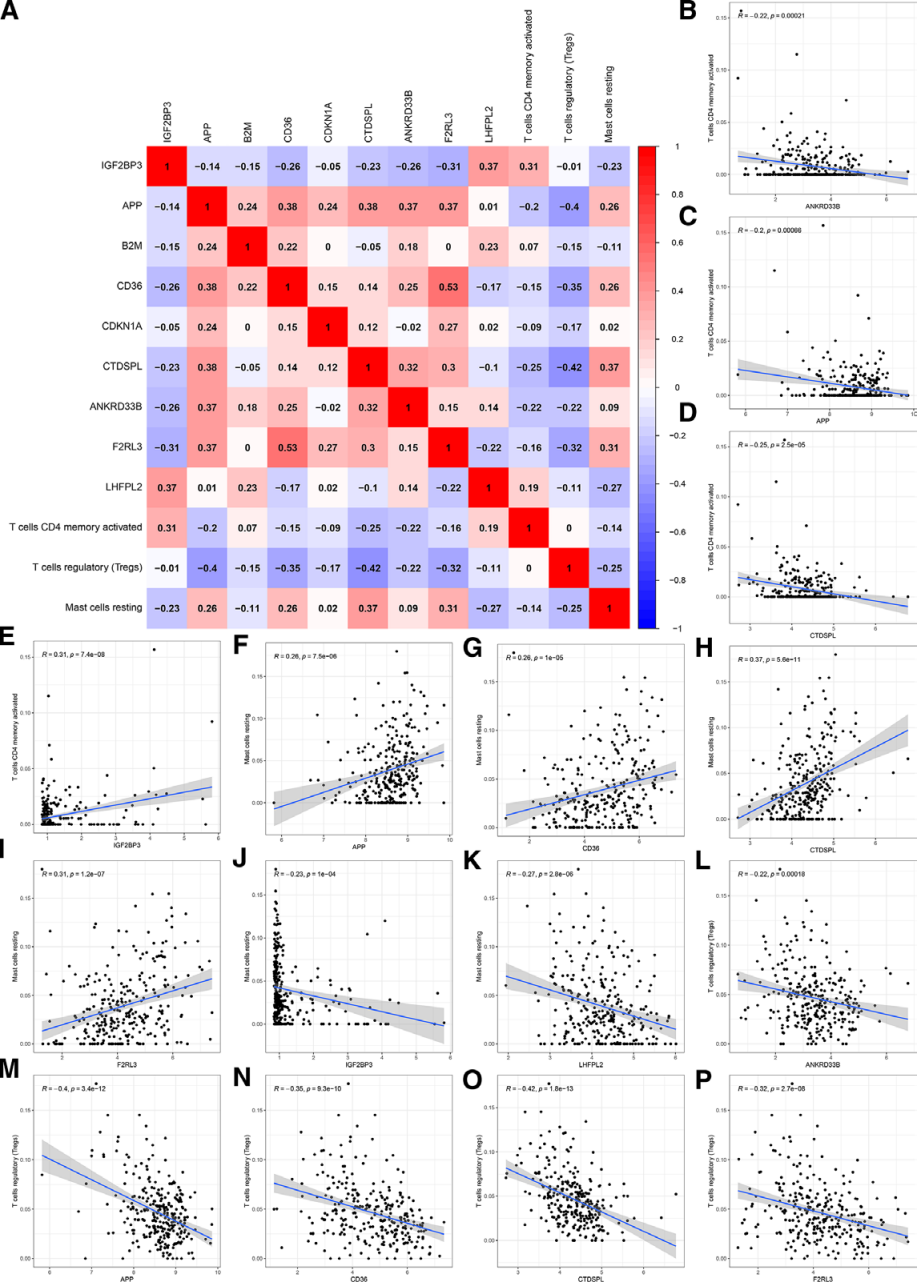
Co-expression analysis of differential immune cells and core mRNAs. The correlation between core mRNAs and differential immune cells. (B–P) The significantly correlated pairs with correlation coefficient > 0.5 and *P* < .001. mRNA = messenger RNA.

The relationship between core mRNAs and differentially expressed immune cells was assessed, revealing substantial correlations with a coefficient >0.5 and a *P*-value <.001, as illustrated in Figure 9. using Pearson correlation analysis. The memory-activated CD4 T cells were negatively correlated with ANKRD33B, APP, and CTDSPL (R = −0.22, R = −0.2, and R = −0.25, respectively), but positively correlated with IGF2BP3 (*R* = 0.31). The ANKRD33B, APP, CD36, CTDSPL, and F2RL3 were both negatively correlated with Tregs (*R* = −0.22, *R* = −0.4, *R* = −0.35, *R* = −0.42, and *R* = −0.32, respectively). The activated mast cells had positive correlations with APP, CD36, CTDSPL, and F2RL3 (*R* = 0.26, *R* = 0.26, *R* = 0.37, and *R* = 0.31), respectively, while demonstrating negative correlations with IGF2BP3 and LHFPL2 (*R* = −0.23 and *R* = −0.27, respectively). Thus, the co-expression analysis of differential immune cells and core mRNAs demonstrates a strong association between core mRNAs and differential immune cells in ccRCC patients.

### 3.7. The relationship between gene prognostic model and tumor mutation burden

**Figure 10.**
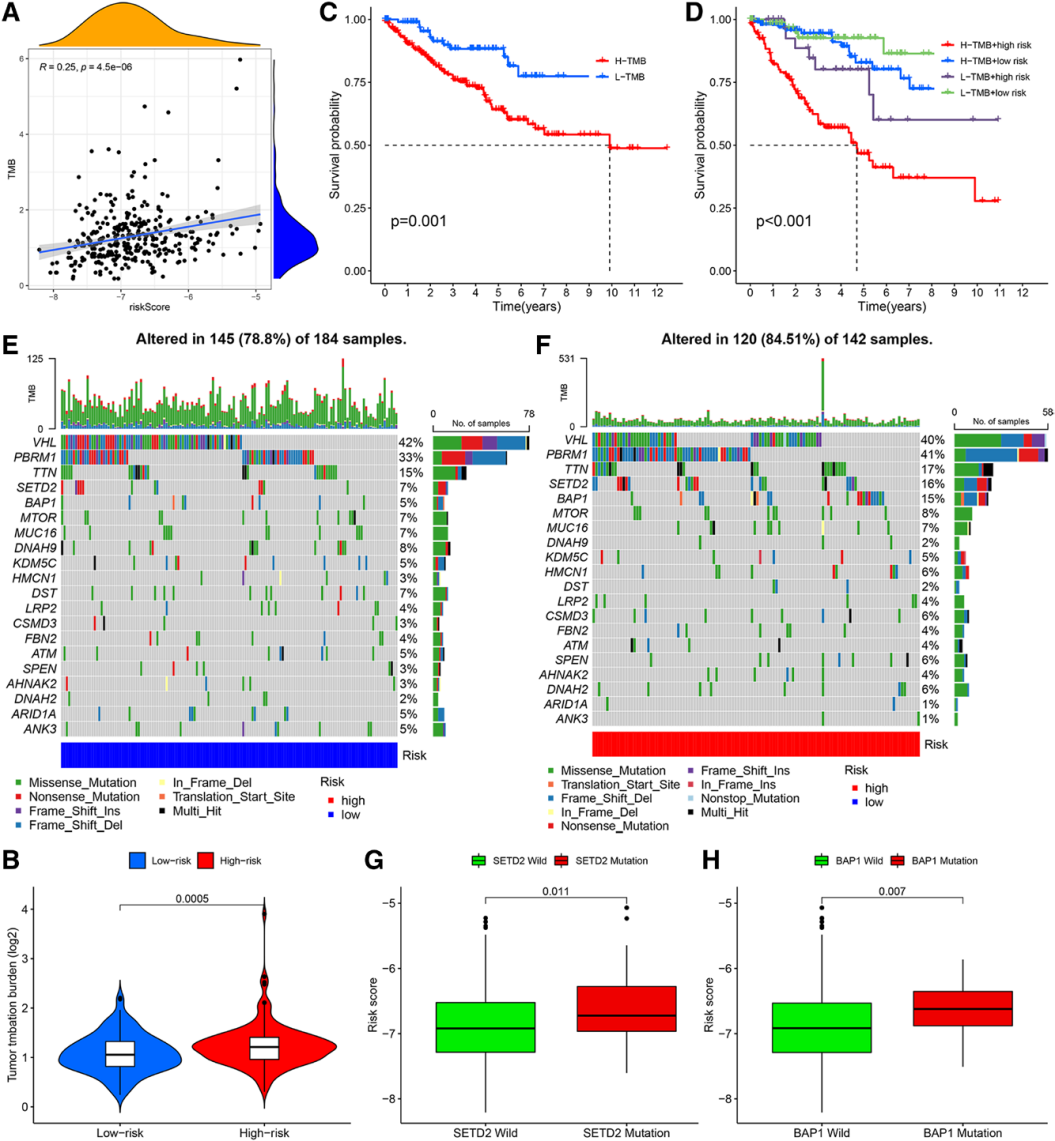
The relationship between the gene prognostic model and tumor mutation burden. (A) Scatter plots of GPM-based risk signature and TMB correlation. (B) TMB comparison between GPM-high and GPM-low. (C) TCGA-KIRC TMB-based Kaplan–Meier survival analysis. (D) Kaplan–Meier survival analysis for 4 TCGA-KIRC cohort groups stratified by TMB and GPM-based risk signature. (E) Waterfall plots show high-risk gene mutation frequencies. A waterfall plot shows genes with high mutation rates in the low-risk group. (G) BAP1 mutation-classified patients had different risk ratings. (H) SETD2 mutation-classified patients had different risk ratings. GPM = genes prognostic model, KIRC = kidney renal clear cell carcinoma, TCGA = The Cancer Genome Atlas, TMB = tumor mutation burden.

First, we found a significant correlation between the risk scores and TMB representing nonsynonymous variants (Fig. 10A). Subsequently, we further analyzed the correlation between the 9 genes prognostic model (GPM) and TMB. There were significant differences in TMB between high and low GPM patients (Fig. 10B). Furthermore, low TMB was found to correlate with good OS (Fig. 10C). We also integrated GPM and TMB to divide all the samples into high TMB/low GPM, low TMB/low GPM, high TMB/high GPM, and low TMB/high GPM groups. Significant differences were observed among all groups (*P* < .0001), and patients in the low TMB/low GPM group exhibited the best OS (Fig. 10D). The top 20 genes with the highest aberrant expression between the high- and low-risk subgroups are shown in Figure 10E and F. Furthermore, patients with BAP1 and SETD2 mutations had higher risk scores (Fig. 10G and H).

### 3.8. Protein levels differential expression and cellular immunofluorescence localization of prognostic model genes

**Figure 11.**
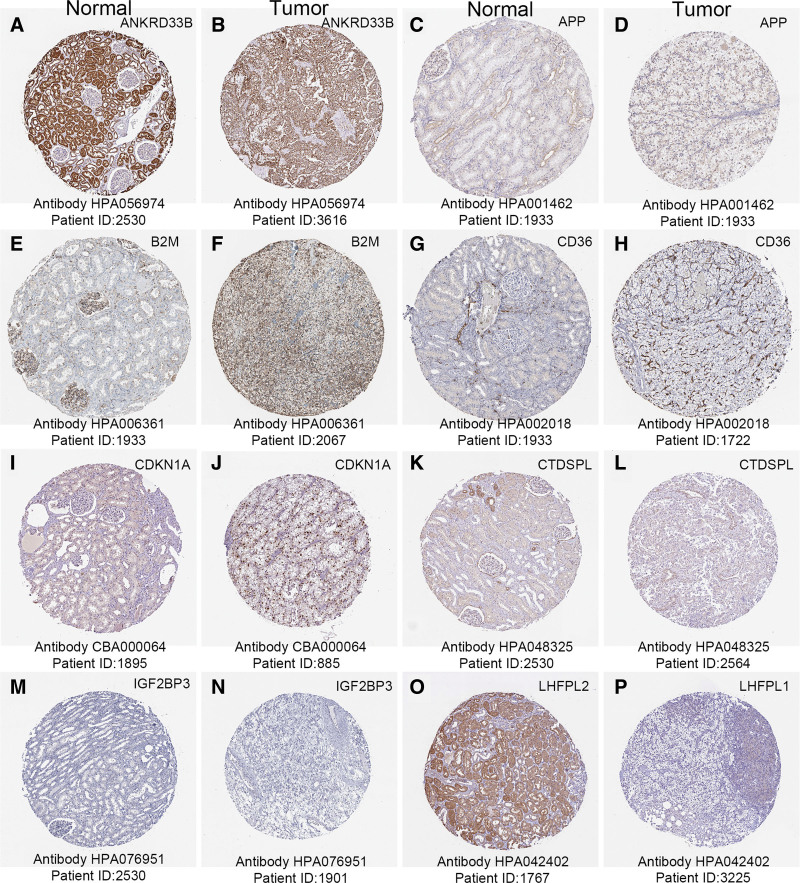
The histological expression of prognostic model genes. The top of the figure indicates the category of tissue specimen. The gene name is in the upper right corner of each image. The antibody type used in immunohistochemistry and the patient ID of tissue specimens are shown at the bottom of each image.

**Figure 12.**
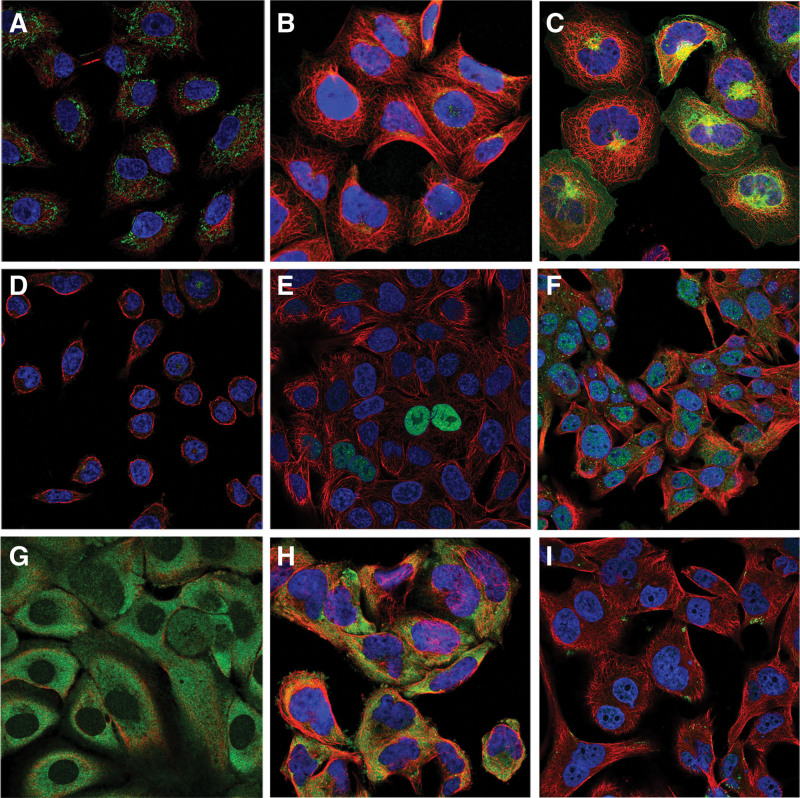
The expression position of prognostic model genes in the cell. (A) ANKRD33B. (B) APP. (C) B2M. (D) CD36. (E) CDKN1A. (F) CTDSPL. (G) F2RL3. (H) IGF2BP3. (I) LHFPL2.

The histological expressions of prognostic model genes in normal and tumor tissues were exhibited in Figure 11. There was a significant expressive difference between these genes in protein levels between normal and renal cancer tissues. Among that, the protein expression of IGF2BP3, B2M, CD36, CDKN1A, ANKRD33B, and LHFPL2 were up-regulated; However, the protein expression of APP and CTDSPL was low in both normal and tumor tissues. And that of F2RL3 was even not detected. The cellular immunofluorescence localization of prognostic model genes was exhibited in Figure 12. ANKRD33B was detected in mitochondria; APP was detected in Golgi apparatus and vesicles; and is predicted to be secreted. B2M is detected in the Golgi apparatus, plasma membrane, and cytosol; and is predicted to be secreted. CD36 is detected in the Golgi apparatus. CDKN1A is detected in the nucleoplasm and nuclear bodies. CTDSPL is detected in vesicles and nucleosomes; F2RL3 is detected in the plasma membrane; IGF2BP3 is detected in the cytosol; LHFPL2 is detected in the plasma membrane, nuclear bodies, and vesicles (Fig. S3, Supplemental Digital Content, http://links.lww.com/MD/N810).

### 3.9. Construction and analysis of subnetwork of prognosis-related mRNAs

**Figure 13.**
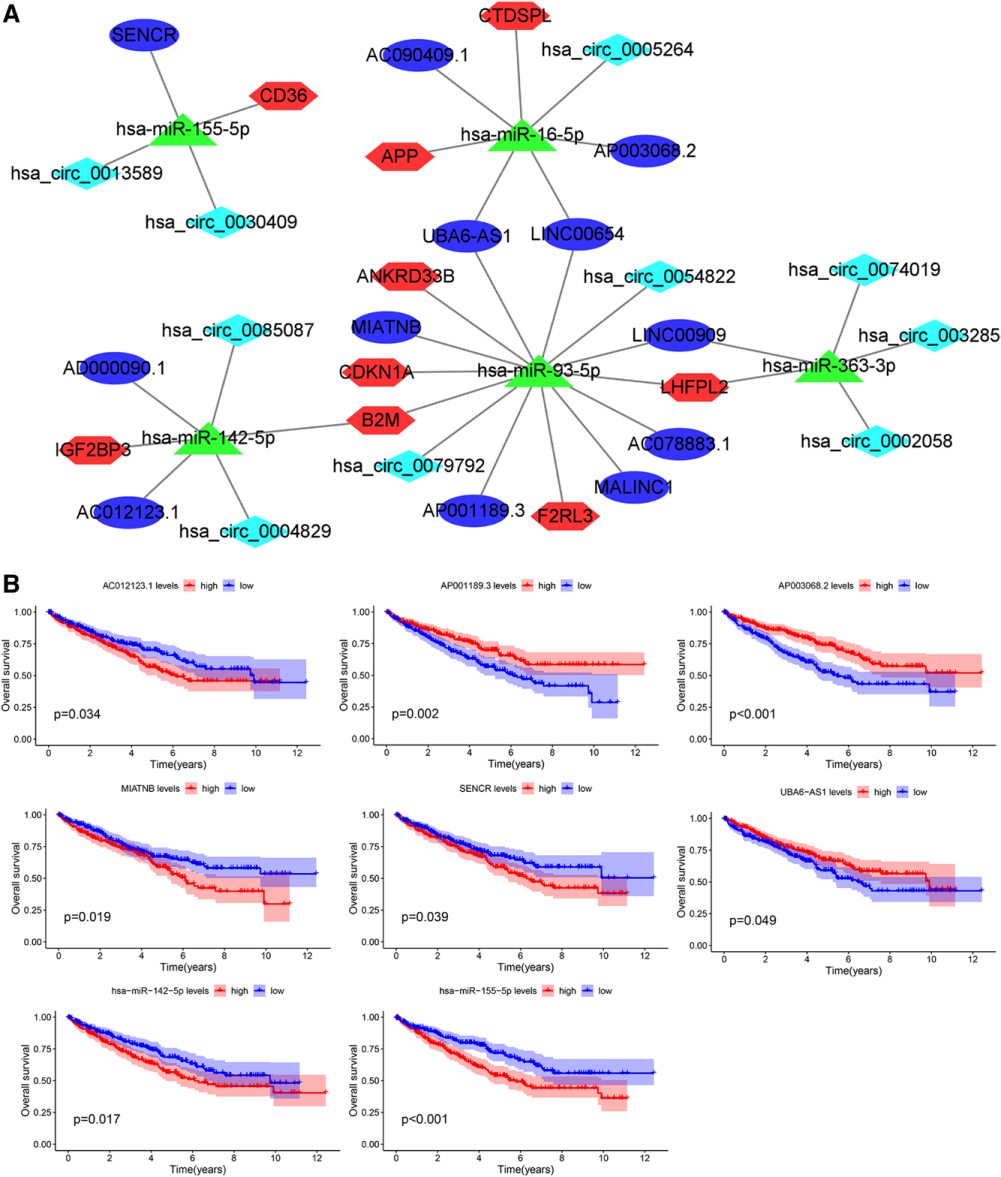
Construction and analysis of subnetwork of prognosis-related mRNAs. (A) Development of the circRNA–lncRNA–miRNA–mRNA subnetwork utilizing mRNAs with predictive significance for survival. Cyan denotes circRNAs, blue signifies lncRNAs, red represents mRNAs, and green indicates miRNAs. (B) Kaplan–Meier survival curves for lncRNAs and miRNAs correlated with overall survival in the subnetwork. lncRNAs = long noncoding RNAs, miRNA = microRNA, mRNA = messenger RNA.

We created a circRNA–lncRNA–miRNA–mRNA subnetwork with 9 prognosis-related mRNAs. As shown in Figure 13A, the subnetwork was composed of 10 circRNA nodes, 12 lncRNA nodes, 5 miRNA nodes, 9 mRNA nodes, and 36 edges. Then, we analyzed the prognosis-related miRNAs and lncRNAs in the subnetwork. Low expression of AC012123.1, MIATNB, SENCR, hsa-miR-142-5p, and hsa-miR-155-5p, and high expression of AP001189.3, AP003068.2, and UBA6-AS1 were associated with good OS (Fig. 13B).

## 4. Discussion

ccRCC is the most common renal cancer and is frequently diagnosed at an advanced stage. It is prone to develop unpredictable metastases even with proper treatment. Exosomes have garnered significant interest in metastatic RCC patients as facilitators of intercellular communication.^[43]^ They are involved in intercellular communications by transferring intracellular molecules into sensitive target cells.^[34,44]^ Exosomes are released into the bloodstream and/or tumor microenvironment and impact tumor-distant metastasis.^[45]^ Both lncRNA and circRNA can act as sponges of miRNA to effectively control the subsequent miRNA posttranscriptional regulation.^[46,47]^ The chemicals, including miRNAs, contained in exosomes and their transmission destinations collaboratively influence cellular processes.^[48]^ Exosomal miRNA can be transmitted to the pre-metastatic niche and influence the gene expression of target cells.^[49]^ Exosomes were found to convey PTEN-targeting miRNAs to metastatic tumor cells, hence enhancing brain metastasis.^[50]^ The exosomal transfer of miR-21 imparts paclitaxel resistance in ovarian cancer cells.^[51]^ Mani et al revealed a new epigenetic regulation of RAD51 in triple-negative breast cancers by miR-214-5P, indicating potential combination therapies utilizing miR-214-5P and olaparib to address HR-proficient triple-negative breast cancers and mitigate racial disparities in treatment results.^[52]^

The ceRNA network theory posits that circRNAs, lncRNAs, and mRNAs compete for miRNA binding through shared miRNA-responsive elements, hence modulating each other’s expression and influencing carcinogenesis and progression.^[53]^ The ceRNA regulation network elucidates the interactions among several RNA types at the molecular level. The lncRNA AFAP-AS1 acting as a miR-155-5p sponge was reported to promote colorectal cancer proliferation by regulating the activity of the mitogen-activated protein kinase1 pathway.^[54]^ Circular RNA TMEM87A regulates gastric cancer cell proliferation and metastasis by increasing ULK1 expression via sponging miR-142-5p.^[55]^ LncRNA TTN-AS1 assists lung adenocarcinoma cell migration, invasion, and epithelial mesenchymal transition and TTN-AS1 may work as a ceRNA that sponges miR-142-5p to regulate the CDK5 expression.^[56]^ Liu et al developed a ceRNA network comprising lncRNAs, miRNAs, and mRNAs, and presented a predictive lncRNA model for ccRCC utilizing the lncRNAs from the ceRNA network.^[36]^ Bai et al revealed a ceRNA network comprising 6 circRNAs, 6 miRNAs, and 10 mRNAs to establish a circRNA signature in ccRCC.^[37]^ However, they exclusively investigated the function of either circRNA–miRNA–mRNA networks or lncRNA–miRNA–mRNA in ccRCC, and a thorough simultaneous analysis of circRNAs, lncRNAs, miRNAs, and mRNAs to establish a predictive ceRNA network for ccRCC has not been conducted. This study involved a thorough analysis of transcriptome data from the TCGA and exoRBase databases, resulting in the development of a predictive circRNA–lncRNA–miRNA–mRNA ceRNA network for ccRCC.

Furthermore, 45 hub mRNAs were identified, followed by the application of Lasso, univariate, and multivariate Cox analysis to construct a predictive model for ccRCC. The 9 genes APP, B2M, CD36, CDKN1A, CTDSPL, ANKRD33B, IGF2BP3, LHFPL2, and F2RL3 were screened to build the prognostic model. We demonstrated that the 9-gene signature serves as an independent prognostic factor for ccRCC patients and confirmed its prediction capacity for survival utilizing the external ccRCC cohort from the E-MTAB-1980 database. The AUC values of the prognostic model for predicting 1, 3, and 5-year survival were 0.730, 0.729, and 0.741, respectively, indicating that the signature had strong efficacy in survival prediction. Consequently, the 9-gene signature can provide insights into the molecular characteristics of ccRCC and may serve as a valuable guide for personalized ccRCC therapy. ccRCC patients frequently acquire resistance to therapies such as radiation and chemotherapy. Identifying biomarkers that forecast treatment results is essential for individualized therapy.

Five pivotal genes in the prognostic signature (B2M, CD36, CDKN1A, IGF2BP3, and F2RL3) are recognized for their involvement in ccRCC. B2M is found on the surface of all nucleated cells and acts to stabilize the major histocompatibility complex class I trimer by non-covalently bonding to the alpha-3 subunit.^[57]^ Extrinsic anchoring of B2M is required for cells, including tumor cells, to express major histocompatibility complex class I and participate in tumor-antigen processing via CD8+ T-cells.^[58]^ Abundant CD8+ TILs and high tumor expression of B2M were good prognostic markers correlated with longer survival in patients with high-stage ccRCC.^[57]^ Overexpressed CD36 was found to correlate with increased visceral adipose tissue and predicted a poor prognosis in ccRCC patients. CD36 is an integral transmembrane glycoprotein detected in various tissues and has a role in high-affinity uptake of long-chain fatty acids.^[59]^ Overexpressed CD36 expression predicts poor prognosis in ccRCC patients.^[60]^ LncRNA SNHG16 promotes ccRCC cell migration and invasion through suppression of CDKN1A.^[61]^ The lncRNA DMDRMR assists IGF2BP3 in stabilizing target genes as a cofactor in an m6A-dependent manner, thus exerting essential oncogenic roles in ccRCC.^[62]^ A high level of F2RL3 was observed to correlate with aggressive features and poor survival in ccRCC.^[63]^

We identified 5 immune cell types, including follicular helper T cells, memory-activated CD4 T cells, and resting mast cells, associated with patient prognosis among 22 immune cell types. We established a Cox proportional hazards regression model, indicating significant clinical value following AUC evaluation. Deng et al^[64]^ and Zhu et al^[65]^ determined the correlation between immune cells and RCC and found that follicular helper T cells were associated with poor prognosis while resting mast cells were positively associated with long-term survival. Genetic ablation of CD36 in Treg cells suppressed tumor growth accompanied by enhancement of antitumor activity in tumor-infiltrating lymphocytes, and a decrease in intratumoral Treg cells without disrupting immune homeostasis. Furthermore, CD36 targeting elicited additive antitumor responses with anti-programmed cell death protein 1 therapy.^[66]^

In this study, we also determined the joint tumor mutation burden and risk score model. Our results showed that the SETD2 and BAP1 mutant-type patients in the high-risk subgroup had a poor prognosis. BAP1 belongs to a subfamily member of deubiquitinating enzymes and is a novel ubiquitin carboxy-terminal hydrolase.^[67,68]^ BAP1 can bind to BRCA1 in the nucleus and increase BRCA1 tumor suppressor activity.^[69]^ It was reported that BAP1-related ceRNA (NEAT1/miR-10a-5p/SERPINE1) promotes kidney cancer cell proliferation and migration.^[70]^ SETD2 is a histone H3 lysine 36 (H3K36) trimethyltransferase and is one of the frequently mutated genes in ccRCC.^[71]^ SETD2 loss perturbs the kidney cancer epigenetic landscape to promote metastasis and engenders actionable dependencies on histone chaperone complexes.^[72]^

Despite our extensive efforts to enhance the representativeness and accuracy of ccRCC-related ceRNAs and the reliability of the prediction through various verification levels, certain limitations persist: Initially, our ceRNA regulation axis was established using bioinformatics research; hence, the conclusion requires validation via in vitro and in vivo experiments. Secondly, we were unable to integrate the results from the co-expression study of ceRNA connection and correlation due to the constraint that the expression data of distinct RNAs were obtained from disparate samples and platforms. Furthermore, several hub circRNAs, lncRNAs, miRNAs, and mRNAs remain devoid of valuable information in ccRCC. Consequently, we confirmed the targeting link across numerous databases and will persist in validating the results in next studies. Future research will broaden the patient group and incorporate supplementary in vivo and in vitro samples to validate the role of the found biomarkers in ccRCC patients, while also examining their function to better assess their clinical applicability and expression processes.

## 5. Conclusions

We established a ccRCC-associated ceRNA regulation network comprising circRNA, lncRNA, miRNA, and mRNA, demonstrating a strong correlation with the prognosis of ccRCC patients. We introduced multiple distinctive potential biomarkers for the diagnosis, treatment, and prognosis assessment of ccRCC patients. We elucidated and examined the posttranscriptional and molecular regulatory mechanisms of ccRCC, identified a novel avenue for comprehending its pathophysiology, and offered more effective targets for diagnostic, treatment, and prognostic prediction.

## Author contributions

**Data curation:** Tao Zhu.

**Formal analysis:** Zhiqiang Wang, Shidong Zhang.

**Investigation:** Tao Zhu, Haizhu FU, Zhiqiang Wang, Shidong Zhang.

**Methodology:** Tao Zhu.

**Software:** Haizhu Fu.

**Supervision:** Zhiqiang Wang, Shanchun Guo.

**Validation:** Shanchun Guo, Shidong Zhang.

**Writing – original draft:** Zhiqiang Wang.

**Writing – review & editing:** Shanchun Guo, Shidong Zhang.

## Supplementary Material


