# Supplementary figures and images for: Identification of exosomal ceRNA networks as prognostic markers in clear cell renal cell carcinoma
Source: Medicine (Baltimore). 2024 Oct 25;103(43):e40167. doi: 10.1097/MD.0000000000040167 (PMC11521039; doi:10.1097/MD.0000000000040167)

Figure S1


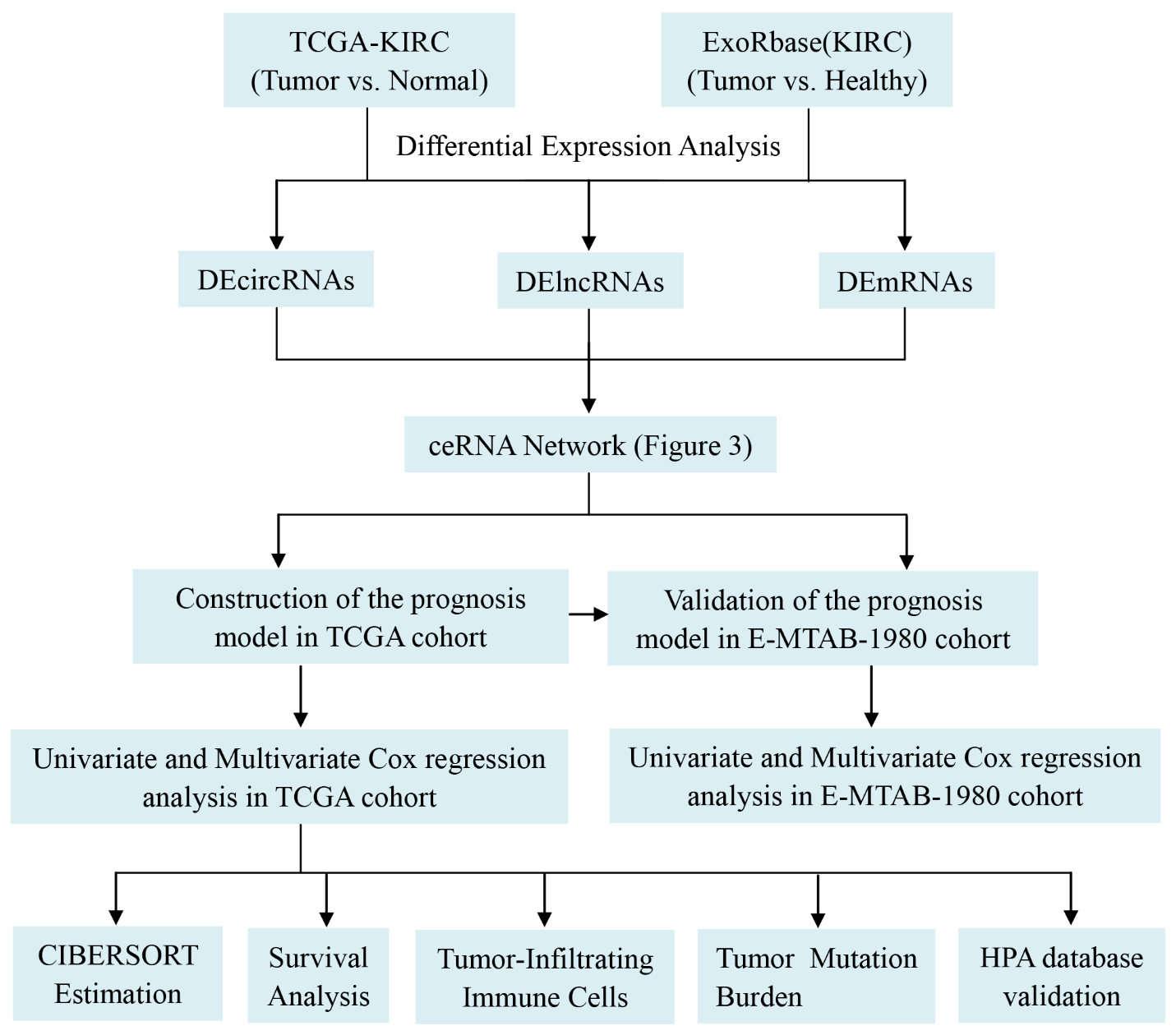


Figure S2


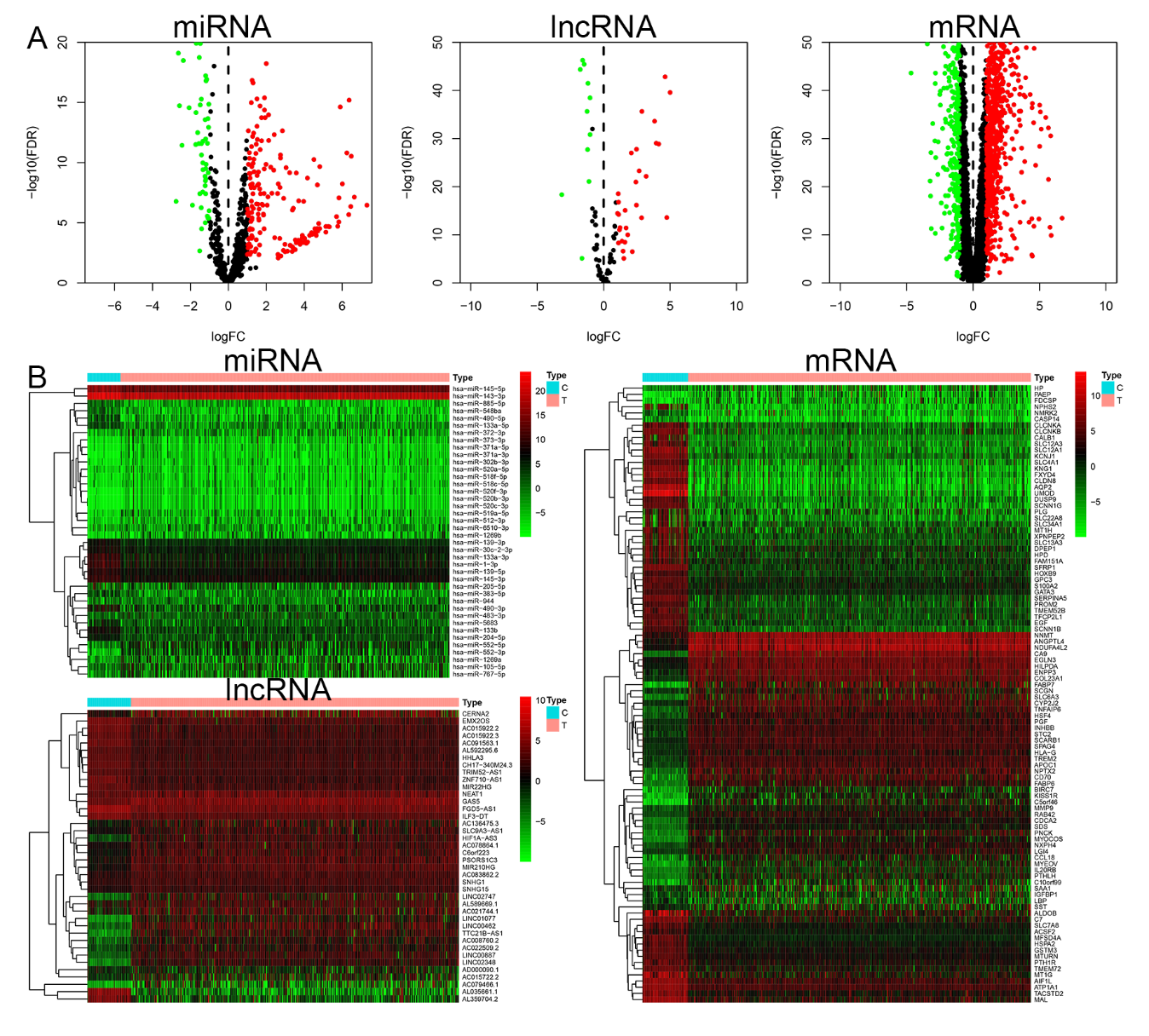


Figure S3


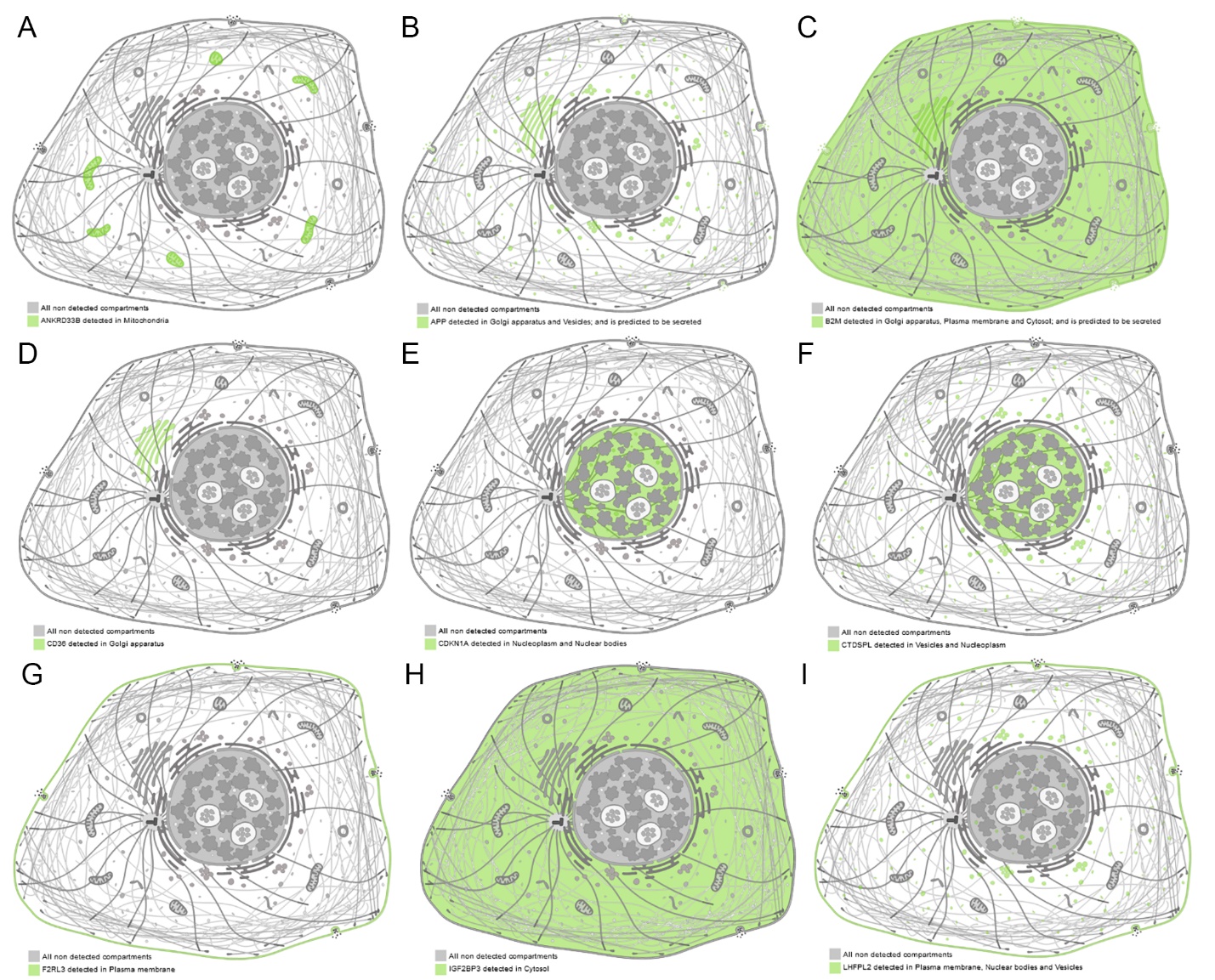

Supplement: Supplementary file 1 [file medi-103-e40167-s001.docx]
